# Activated spinal astrocytes are involved in the maintenance of chronic widespread mechanical hyperalgesia after cast immobilization

**DOI:** 10.1186/1744-8069-10-6

**Published:** 2014-01-24

**Authors:** Mika Ohmichi, Yusuke Ohmichi, Hitoshi Ohishi, Takahiko Yoshimoto, Atsuko Morimoto, Yuqiang Li, Hiroki Sakurai, Takashi Nakano, Jun Sato

**Affiliations:** 1 Department of Anatomy, Aichi Medical University, Aichi 480-1195, Japan; 2 Research Institute of Environmental Medicine, Nagoya University, Aichi 464-8601, Japan; 3 Multidisciplinary Pain Center, Aichi Medical University, Aichi 480-1195, Japan; 4 Faculty of Health and Medical Sciences, Tokoha University, Hamamatsu 431-2102, Japan; 5 Center for Animal Research and Education, Nagoya University, Aichi 464-8601, Japan

**Keywords:** Cast immobilization, Chronic post-cast pain, Widespread hyperalgesia, Complex regional pain syndrome (CRPS) Type I, Microglia, Astrocytes

## Abstract

**Background:**

In the present study, we examined spinal glial cell activation as a central nervous system mechanism of widespread mechanical hyperalgesia in rats that experienced chronic post-cast pain (CPCP) 2 weeks after cast immobilization. Activated spinal microglia and astrocytes were investigated immunohistologically in lumbar and coccygeal spinal cord segments 1 day, 5 weeks, and 13 weeks following cast removal.

**Results:**

In the lumbar cord, astrocytes were activated after microglia. Astrocytes also were activated after microglia in the coccygeal cord, but with a delay that was longer than that observed in the lumbar cord. This activation pattern paralleled the observation that mechanical hyperalgesia occurred in the hindleg or the hindpaw before the tail. The activating transcription factor 3 (ATF3) immune response in dorsal root ganglia (DRG) on the last day of cast immobilization suggested that nerve damage might not occur in CPCP rats. The neural activation assessed by the phosphorylated extracellular signal-regulated kinase (pERK) immune response in DRG arose 1 day after cast removal. In addition, L-α-aminoadipate (L-α-AA), an inhibitor of astrocyte activation administered intrathecally 5 weeks after cast removal, inhibited mechanical hyperalgesia in several body parts including the lower leg skin and muscles bilaterally, hindpaws, and tail.

**Conclusions:**

These findings suggest that activation of lumbar cord astrocytes is an important factor in widespread mechanical hyperalgesia in CPCP.

## Background

Peripheral nerve injury can provoke chronic neuropathic pain that persists longer than the actual nerve injury. Indicators of pain have been reported in areas other than those attributed to the injured nerve, such as contralateral regions [1-3]. Studies of chronic pain occurring without clear nerve injury, as with complex regional pain syndrome (CRPS) Type I [4], also noted chronic widespread pain (CWP) in which the affected area spread far beyond the region of the initial injury and increased over time [5-7]. What is especially puzzling is that the triggers for CWP can be minor injuries [4,8] or limb immobilization [4,9-11], thus the severity of the symptom is clearly disproportionate to the cause. This suggests that CWP operates on mechanisms that are different from those for neuropathic pain. However, research on chronic pain mechanisms has focused on neuropathic pain models [2,3,12,13], and research using animal models for chronic pain arising due to tissue damage distinct from nerve injury (such as muscle damage, fracture, or ischemic reperfusion) has only just begun [14-17].

Therefore, we have developed a chronic post-cast pain (CPCP) model in rats with unilateral cast immobilization of the hindlimb to examine the mechanisms mediating CWP [18]. The hyperalgesic behavior observed in this model appeared on the immobilized side immediately after cast removal, but also showed a delayed expansion to the contralateral side, and eventually expanded to the tail [18,19]. Blocking the sciatic nerve of the immobilized limb with lidocaine 24 h after cast removal temporarily abolished the mechanical hyperalgesia bilaterally in CPCP rats [18], suggesting that sensory inputs originating from the immobilized hindlimb contributed to the bilateral hyperalgesia. In contrast, when the sciatic nerve block was performed 3–8 weeks after cast removal, mechanical hyperalgesia in the contralateral hindpaw was not altered. Based on these results, we hypothesized that peripheral nociceptive signals induce changes at the central level (central sensitization), and this phenomenon leads to chronic pain.

Research using animal models of nerve injury has implicated activated spinal microglia in hyperalgesia observed on the side ipsilateral to the nerve injury, whereas activated spinal astrocytes were implicated in the persistence of such pain [20-27]. Furthermore, research using animal models of inflammation has implicated activated spinal glial cells in bilateral pain [28-30]. These reports suggest that activated spinal glial cells also contribute to the widespread hyperalgesia observed in CPCP model rats; therefore, we investigated activated spinal microglia and astrocytes in the CPCP model. More specifically, we examined activated microglia and astrocytes in the lumbar cord and coccygeal cord immunohistologically at three time points following cast removal to determine whether a glial activation inhibitor could inhibit CWP.

## Results

### Mechanical hyperalgesia spreads chronically beyond the cast-immobilized area

**Figure 1:**
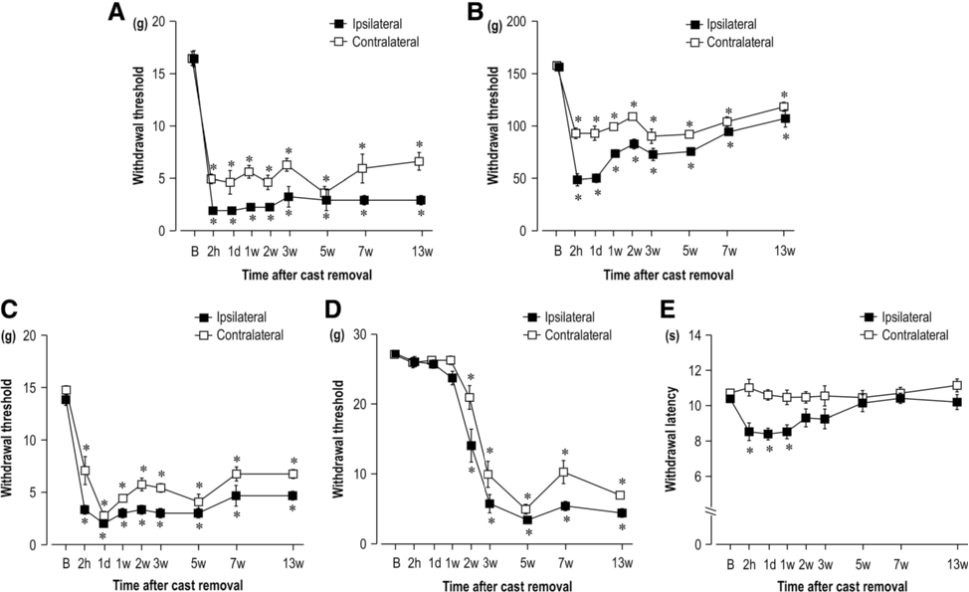
**Time courses of the changes in mechanical and heat hyperalgesia in chronic post-cast pain (CPCP) rats. (A–D)** Time courses of the changes in mechanical pain threshold in the bilateral calf skin **(A)**, calf muscle **(B)**, hindpaws **(C)**, and both sides of the tail **(D)** after cast removal (n = 6). **(E)** Time course of the change in heat withdrawal latency in both hindpaws (n = 11). The horizontal axis indicates measurement time points (B: before cast, 2 h: 2 hours, 1 d: 1 day, w: weeks). Data are presented as the mean ± SEM. One-way ANOVAs and Dunnett’s post hoc tests identified significant threshold changes in all tested body parts after cast removal. *p < 0.05, compared with B value.

The mechanical pain thresholds of the calf skin (Figure 1A), calf muscle (Figure 1B), hindpaws (Figure 1C), and tail (Figure 1D) in the CPCP rats were measured for 13 weeks following cast removal. The mechanical pain thresholds for the calf skin and calf muscle just below the cast-immobilized area showed a significant, rapid decline in the immobilized side thresholds 2 h after cast removal. The mechanical pain thresholds in both hindpaws showed a significantly slower rate of decline than the calf thresholds 2 h after cast removal. The mechanical pain threshold for the tail also decreased significantly 2 weeks after cast removal, and the rate of decline was even slower than that for the calf or hindpaw. These instances of mechanical hyperalgesia were sustained for periods lasting 13 weeks or longer. These results indicate a trend that is similar to that observed in our previous study assessing mechanical hyperalgesia by the number of withdrawal responses [18]. Heat pain latency was also measured until week 13 after cast removal, which was conducted in a manner similar to that for the mechanical pain thresholds (Figure 1E). A decline in the heat pain latency that was approximately 80% of the baseline value was observed on the ipsilateral side from 2 h after cast removal until week 1. This decline in latency reached the baseline level by the second week following cast removal. Unlike the mechanical hyperalgesia, heat hyperalgesia occurred on only one side and recovered sooner. These results demonstrate that only mechanical hyperalgesia spreads beyond the cast-immobilized area.

### Differences in the time course of activation between spinal microglia and astrocytes in CPCP rats

**Figure 2:**
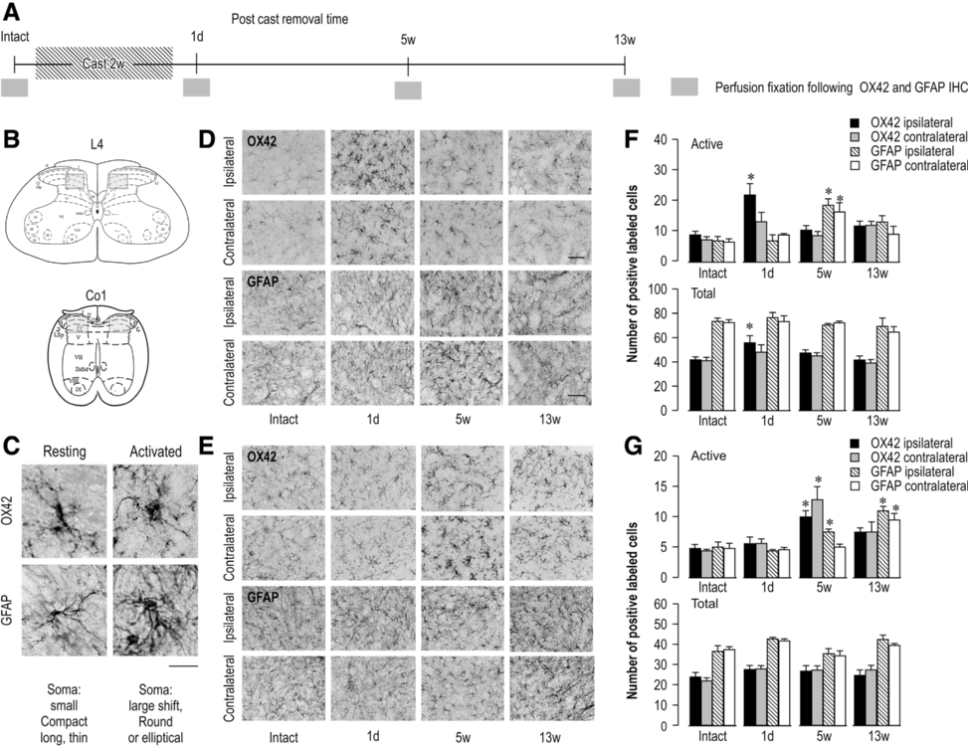
**Glial cell activation spread beyond the immobilized hindlimb. (A)** Schematic time-line for fixation and immunohistochemistry (1 d: 1 day, w: weeks). **(B)** Schematic illustration of the spinal cord segments, 4th lumbar (L4) and 1st coccygeal (Co1). Predefined square areas including lamina II–IV designate the area of quantitative analysis of microglia and astrocyte activation. **(C)** Glial staining indicating glial activation. Quantitative analysis was performed by a blindfolded independent observer using a counter. Scale bar = 20 μm. **(D, E)** Temporal patterns of glial cells in L4 **(D)** and Co1 **(E)** using OX42 and GFAP staining. Scale bar = 50 μm. **(F, G)** Number of positive labeled glial cells with OX42 and GFAP staining in L4 **(F)** and Co1 **(G)** segments. Upper graph shows the number of positive labeled activated glial cells; lower graph shows the total number of positive labeled glial cells. Results shown are the mean ± SEM (n = 4–6). *p < 0.05 compared with the intact group (one-way ANOVA followed by Dunnett’s post hoc test).

To quantitatively analyze spinal microglia and astrocyte activation in the CPCP model, the 4th lumbar (L4) and 1st coccygeal (Co1) spinal cord segments were removed at 1 day, 5 weeks, and 13 weeks after cast removal. These segments were examined with an immunohistological analysis using the microglial marker, OX42, and the astrocyte marker, glial fibrillary acidic protein (GFAP; Figure 2A, B). Figure 2C illustrates the criteria for activated glial cells. As the images indicate, resting glial cells have cell bodies that are small and elongated with long protrusions, whereas activated glial cells have expanded cell bodies with a characteristic round shape. Based on these criteria, panels D and F of Figure 2 show the temporal changes in the activated cell count and total cell count among L4 spinal glial cells in the CPCP rats, respectively. One day after cast removal, the activated cell count for ipsilateral microglia (OX42-positive) increased. This change was statistically significant when compared with that for intact rats on the ipsilateral side of the L4 spinal cord (p < 0.01). The total cell count also significantly increased when compared with that for intact rats on the ipsilateral side (p < 0.05). Five weeks after cast removal, the cell count for activated astrocytes (GFAP-positive) on the ipsilateral side increased to about 2.7 times the value for intact rats and about 2.5 times the value for the contralateral side. These changes were both statistically significant (p < 0.01). We observed no significant change in the total cell count for astrocytes. Panels E and G in Figure 2 illustrate the temporal changes in the activated and total cell counts among Co1 spinal cord glial cells, respectively. The activated cell count for Co1 cord microglia (OX42-positive) increased 5 weeks after cast removal. This change was statistically significant when compared with that for intact rats on the immobilized side 1 day after cast removal (p < 0.01), and that for the contralateral side (p < 0.01). No significant change in the total cell counts was observed. The activated cell counts for Co1 spinal cord astrocytes (GFAP-positive) were approximately 1.5 times greater on the ipsilateral side (p < 0.05), but without significant change on the contralateral side 5 weeks after cast removal. Thirteen weeks after cast removal, the activated cell counts for the Co1 spinal cord astrocytes on both sides increased significantly (for both, p < 0.01). We observed no significant change in the total cell count for the coccygeal cord astrocytes. These results show that activation of lumbar spinal cord microglia began at the onset of mechanical hyperalgesia in the hindpaws and calves, whereas activation of the lumbar spinal astrocytes began during the later maintenance phase. These findings also show a delayed appearance in the coccygeal cord, which paralleled the appearance of mechanical hyperalgesia in the tail, where it appears relatively late. That is to say, these results suggest that microglia are activated at the onset of mechanical hyperalgesia while astrocytes are activated in the maintenance phase.

### Activated lumbar spinal astrocytes are involved in the maintenance of mechanical hyperalgesia

**Figure 3:**
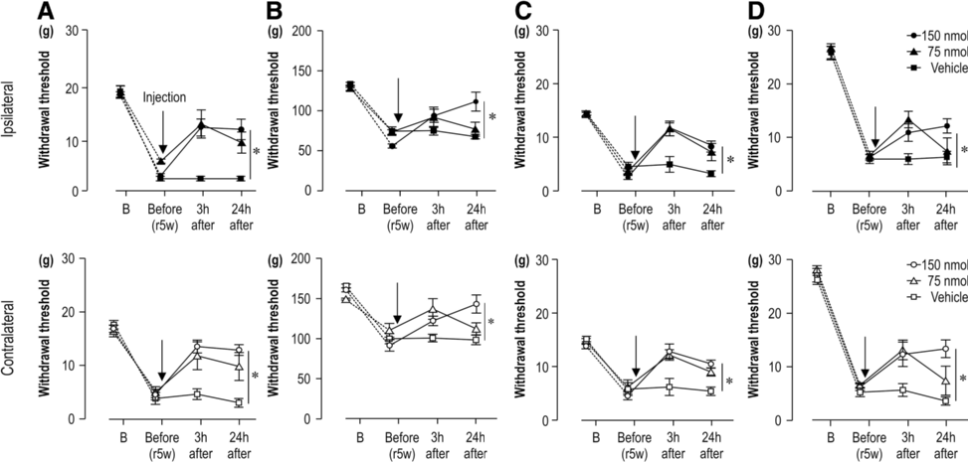
**Intrathecal application of L-α-AA inhibits widespread mechanical hyperalgesia.** Graphs show the time courses of the changes in bilateral mechanical hyperalgesia after two doses (75 and 150 nmol) of L-α-AA administration as compared to the vehicle (PBS) in the calf skin **(A)**, calf muscle **(B)**, hindpaw **(C)**, and both sides of the tail **(D)** (n = 5–6, mean ± SEM). L-α-AA (75 nmol, 150 nmol) or vehicle (PBS) was injected intrathecally (arrows) just after measurements of baseline mechanical threshold (Before) at 5 weeks after cast removal, and the measurements were repeated 3 h and 24 h later. *p < 0.05 as compared with each vehicle treatment group by a mixed-design two-way ANOVA.

To determine whether astrocyte activation was involved in the maintenance of mechanical hyperalgesia in the CPCP rats, and whether lumbar spinal cord microglia were activated at the onset of mechanical hyperalgesia in the hindpaw, L-α-aminoadipate (L-α-AA), a specific astroglial toxin that leads to astrocyte degeneration [22,31,32], was administered intrathecally after measuring the mechanical thresholds 5 weeks after cast removal. Figure 3 shows the time courses of mechanical withdrawal thresholds in the calf skin (Figure 3A), calf muscle (Figure 3B), hindpaw (Figure 3C), and tail (Figure 3D) of CPCP rats after two doses (75 and 150 nmol) of L-α-AA administration. The mixed-design two-way analysis of variance (ANOVA) indicated that the decreased mechanical threshold in the calf skin was significantly reversed bilaterally after administration of 150 nmol of L-α-AA [ipsilateral: treatment F(1,9) = 25.5, p < 0.001; time F(3,27) = 442.6, p < 0.001; treatment × time F(3,27) = 13.5, p < 0.001; contralateral: treatment F(1,9) = 7.0, p < 0.05; time F(3,27) = 38.5, p < 0.001; treatment × time F(3,27) = 11.1, p < 0.001; Figure 3A]. The inhibitory effect of 150 nmol of L-α-AA on hyperalgesia was also observed in the calf muscle [ipsilateral: treatment F(1,9) = 3.8, p > 0.05, time F(3,27) = 48.2, p < 0.001, treatment × time F(3,27) = 11.0, p < 0.001; contralateral: treatment F(1,9) = 6.3, p < 0.05, time F(3,27) = 44.4, p < 0.001, treatment × time F(3,27) = 8.3, p < 0.01; Figure 3B], hindpaws [ipsilateral: treatment F(1,9) = 6.7, p < 0.05, time F(3,27) = 76.3, p < 0.001, treatment × time F(3,27) = 14.7, p < 0.001; contralateral: treatment F(1,9) = 6.95, p < 0.05, time F(3,27) = 38.5, p < 0.001, treatment × time F(3,27) = 11.1, p < 0.001; Figure 3C], and tail [ipsilateral: treatment F(1,9) = 4.9, p > 0.05, time F(3,27) = 195.4, p < 0.001, treatment × time F(3,27) = 5.7, p < 0.01; contralateral: treatment F(1,9) = 6.9, p < 0.05, time F(3,27) = 215.2, p < 0.001, treatment × time F(3,27) = 11.6, p < 0.001; Figure 3D]. The lower dose (75 nmol) of L-α-AA administration also reversed the mechanical hyperalgesia, but the duration of the effect was shortened, indicating the L-α-AA effect occurred in a dose dependent manner.

**Figure 4:**
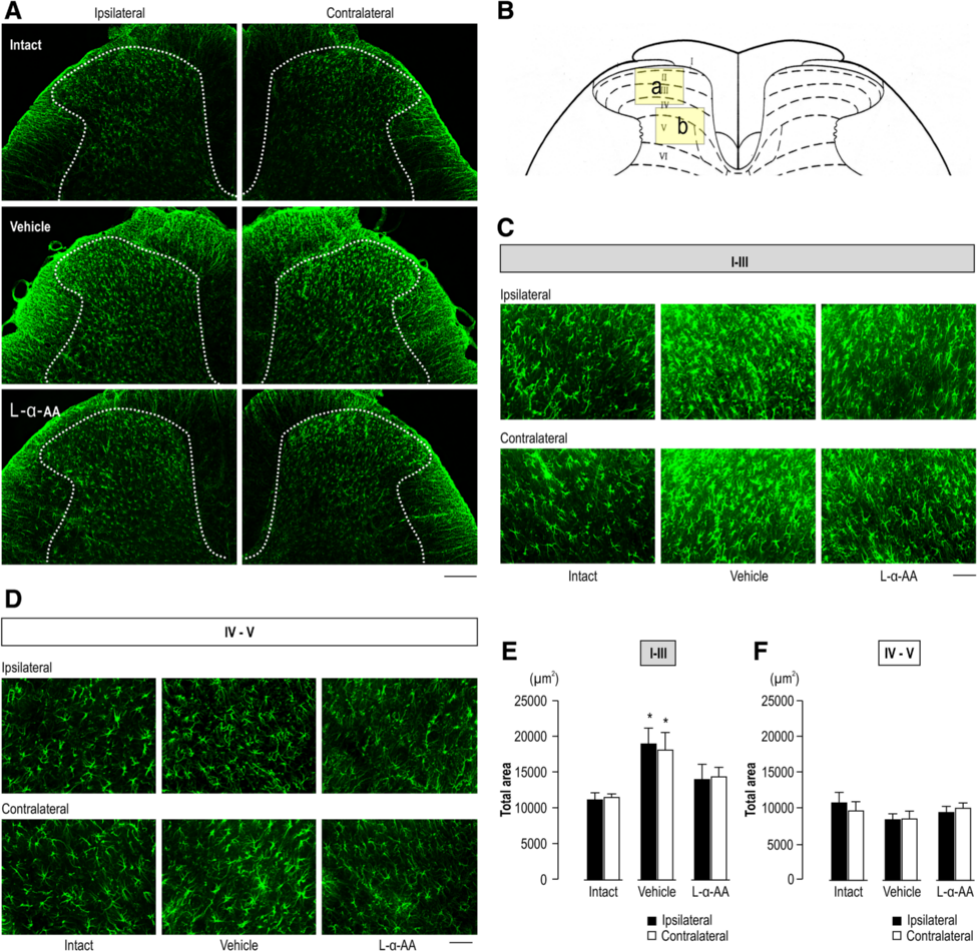
**Intrathecal application of L-α-AA inhibits bilateral astrocyte activation in CPCP rats. (A)** Low-magnification images of GFAP immunoreactivity in L4 spinal segments. Scale bar = 200 μm. L-α-AA (150 nmol) or vehicle (PBS) was injected intrathecally 5 weeks after cast removal, and the former clearly decreased bilateral GFAP immunoreactivity. **(B)** Schematic illustration of L4. The predefined square area including the superficial dorsal horn (lamina I-III) (a) and deep dorsal horn (IV–V) (b) designates the area of quantitative analysis of GFAP astrocyte immunoreactivity. **(C)** High-magnification images of GFAP immunoreactivity in L4 lamina I–III. Scale bar = 50 μm. **(D)** High-magnification images of GFAP immunoreactivity in L4 IV–V. Scale bar = 50 μm. **(E)** The total area of GFAP immunoreactivity in L4 lamina I–III (n = 5). **(F)** Total area of GFAP immunoreactivity in L4 lamina IV–V (n = 5–6). Values are the mean ± SEM. *p < 0.05 as compared with the intact (without cast immobilization) group (unpaired *t*-test).

Figure 4 shows changes in the number of activated lumbar spinal astrocytes in CPCP rats with elevated mechanical thresholds after L-α-AA (150 nmol) administration. In the vehicle-administered CPCP rats, activated astrocyte cell count was significantly increased in the superficial dorsal horn bilaterally (Figure 4A, C, and E). No change was observed in the deep dorsal horn (Figure 4A, D, and F). L-α-AA administration significantly reduced the activated cell count in the superficial dorsal horns bilaterally (Figure 4A, C, and E). These results suggest that activated astrocytes in the lumbar spinal cord are involved in the maintenance of mechanical hyperalgesia in CPCP rats.

### L-α-AA has no impact on activation of OX42-positive microglia at Co1 spinal cord

**Figure 5:**
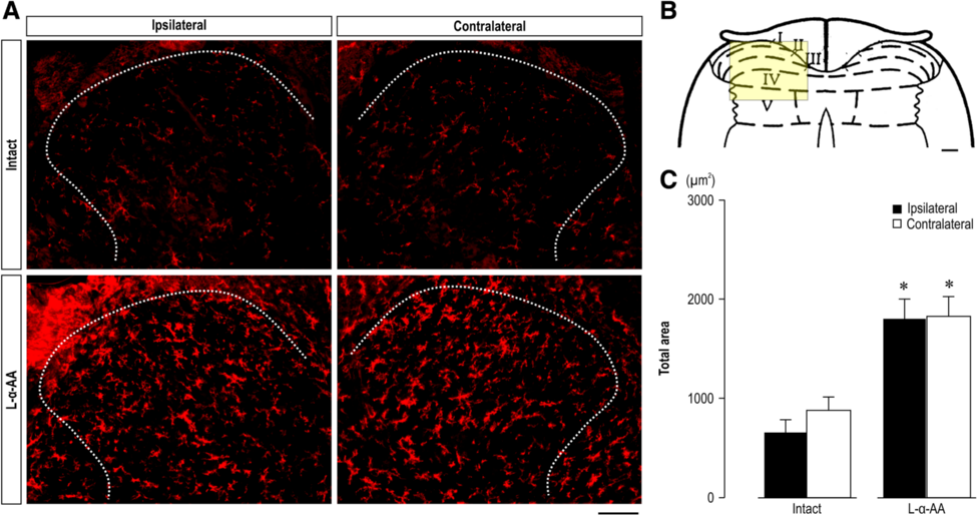
**Intrathecal application of L-α-AA does not affect microglia activation in Co1 spinal cord of CPCP rats. (A)** Low-magnification images of OX42 immunoreactivity in Co1 spinal segments. Scale bar = 200 μm. In the lower images, L-α-AA (150 nmol) was injected intrathecally 5 weeks after cast removal, and this treatment did not affect increased OX42 immunoreactivity. **(B)** Schematic illustration of Co1. The predefined inside square area including lamina I–IV designates the area of quantitative analysis of OX42 microglia immunoreactivity. **(C)** The total area of OX42 immunoreactivity in Co1 in intact rats and L-α-AA-treated CPCP rats. *p < 0.05 as compared with the intact group (ANOVA followed by Dunnett’s post hoc test).

The elevated mechanical threshold for the tail after L-α-AA administration was lower compared to other body parts 5 weeks after cast removal (Figure 3). Therefore, we examined whether Co1 microglia were activated at this time by immunohistologically analyzing the expression of OX42 (Figure 5). Compared to the untreated (intact) group, the activated cell counts for Co1 microglia in the L-α-AA group were 2.8 times greater on the ipsilateral side (p < 0.005) and 2.1 times greater on the contralateral side (p < 0.005) of CPCP rats. This finding suggests that intrathecally administered L-α-AA did not affect activation of OX42-positive microglia at Co1.

### Two-week cast immobilization of one hindpaw did not cause clear nerve damage

**Figure 6:**
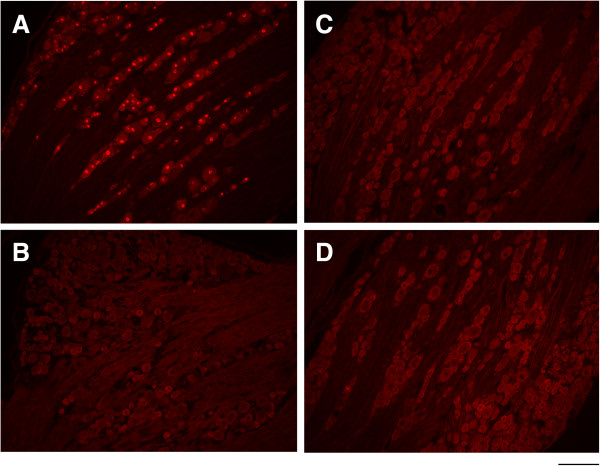
**Clear neuronal injury exhibited by ATF3 immunoreactivity does not occur in the CPCP rat.** These pictures show ATF3 immunoreactivity in the L4 dorsal root ganglion 3 days after complete sciatic nerve transection **(A)**, in the intact (control) group **(B)**, at the final day of cast immobilization (ipsilateral side) **(C)**, and at the final day of cast immobilization (contralateral side) **(D)**. A substantial number of stained cells were seen only in the sciatic nerve transected sample **(A)**, but not in the intact **(B)** or CPCP groups **(C, D)**. Scale bar = 200 μm.

We examined whether 2-week cast immobilization induced nerve damage. For this analysis, the L3, L4, and L5 dorsal root ganglia (DRG), which innervated the spinal cord segments for the cast immobilization site, were removed and immunohistologically examined with activating transcription factor 3 (ATF3), a specific marker of nerve injury (Figure 6, Table 1). We observed that ATF3-positive cells accounted for 56.78% of the total cell count 3 days after sciatic nerve transection (Figure 6A, Table 1). This change was statistically significant (p < 0.05) compared to the intact group (Figure 6B, Table 1). On the other hand, ATF3-positive cell counts were small in L3, L4, and L5 DRG of CPCP rats, and were not significantly different from the 0.33% observed in untreated intact rats (Figure 6C, D). These results suggest that 2-week cast immobilization of one hindpaw did not cause nerve damage.

**Table 1:** ATF3 immunoreactivity in DRG at 2-week cast immobilization

	**Intact**	**SNT**	**CPCP ipsilateral**	**CPCP contralateral**
L3 DRG (n)	―	―	1.64 ± 0.37% n.s. (6)	0.39 ± 0.15% n.s. (6)
L4 DRG (n)	0.33 ± 0.12% (6)	56.78 ± 2.48% # (6)	1.30 ± 0.53% n.s. (5)	0.12 ± 0.08% n.s. (5)
L5 DRG (n)	―	―	0.85 ± 0.16% n.s. (6)	0.65 ± 0.22% n.s. (6)

DRGs were harvested 3 days after SNT, week 2 during cast immobilization. All values are means ± SEM (n = 5–6). #p < 0.05 as compared to the intact value, n.s.: no significance to the intact value (Dunnett’s post hoc test). DRG: dorsal root ganglion; SNT: sciatic nerve transection; CPCP: chronic post-cast pain.

### Apparent neural activation in dorsal root ganglion was induced after cast removal

**Figure 7:**
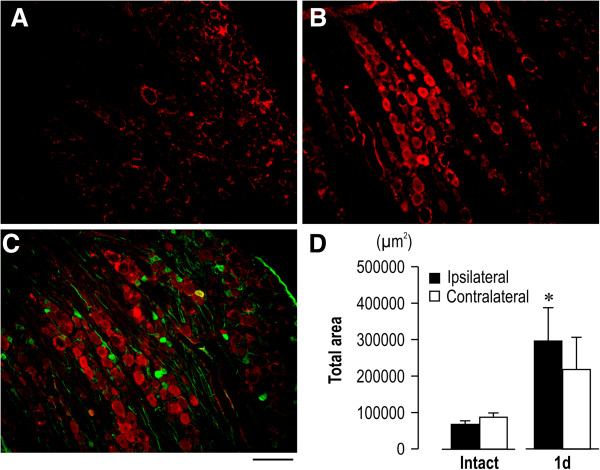
**The number of pERK-positive cells in dorsal root ganglion (DRG) is increased after cast removal. (A, B)** pERK immunoreactivity in L4 DRG of the intact group **(A)** and 1 day after cast removal **(B)**. **(C)** Double staining of pERK and SP. **(D)** The quantification of the total area of pERK immunoreactivity in both L4 DRGs. 1 d: 1 day after cast removal. Values are the mean ± SEM. *p < 0.05 as compared with the intact group (unpaired *t*-test).

Finally, we examined whether 2-week cast immobilization induced neural activation in DRG. For this analysis, the L4 DRG was removed and immunohistologically examined with phosphorylated extracellular signal-regulated kinase (pERK), a marker of neural activation. The activated cell counts for the L4 DRG (pERK-positive) were approximately 3.2 times greater on the ipsilateral side (p < 0.05), but without significant change on the contralateral side 1 day after cast removal (Figure 7A, B, and D). Furthermore, the pERK immunoreactivity was rarely colocalized with the Substance P (SP) immunoreactivity (Figure 7C). These results indicate that the cast removal induced apparent activation in ipsilateral large DRG neurons.

## Discussion

In the present report, we studied the temporal changes in mechanical and heat pain thresholds in CPCP rats over a 13-week period following cast removal. After cast removal, mechanical hyperalgesia spread beyond the immobilized hindlimb with an increasing temporal delay leading away from the immobilized site. These observations are consistent with our previous results that cast immobilization induced local inflammatory changes and spontaneous pain in the immobilized hindlimb, and long-lasting mechanical hyperalgesia in the immobilized limb, contralateral hindlimb, and the tail [18]. The present results further demonstrate that cast immobilization can induce heat hyperalgesia in the immobilized side. Birklein et al. [33] and Sieweke et al. [34] reported in their clinical studies that in patients with acute pain, both heat and mechanical hyperalgesia were observed, whereas in patients with CRPS Type I, only mechanical hyperalgesia was observed. In light of this difference in clinical signs, the authors demonstrated that pain in CRPS Type I is likely related to plastic changes in the central nervous system (CNS). The overlapping features between CRPS and our CPCP rat model suggest that plastic changes in CNS play a role in the pain enhancing mechanisms of the CPCP model.

In the present experiment, we focused on pain enhancing mechanisms through activation of spinal glial cells, which have been reported as one form of plastic abnormality in the CNS [20,22,23,25,35-37]. Studies using nerve injury models have described spinal microglia activation at the onset of hyperalgesia, whereas others have indicated that spinal astrocyte activation helps to maintain such pain [20,22,23,25,35-37]. Reports from studies using inflammatory models have implicated activated spinal glia in bilateral pain as well [28-30,38]. Therefore, we performed a bilateral immunohistological examination of activated spinal microglia and astrocytes in the CPCP model in multiple segments of the lumbar and coccygeal spinal cords at three time points to match the temporal progression of the hyperalgesia after cast removal. Immunohistological analysis with OX42 and GFAP showed activation of spinal microglia 1 day after cast removal, which disappeared 5 weeks after cast removal. However, when microglia activation subsided, astrocyte activation increased. This transition in glial cell activation was observed with a time delay in the coccygeal spinal cord.

It has been shown that the activation of microglia in the spinal cord is accompanied by an increase in the number of microglial cells (proliferation) under several neuropathic pain conditions [20,39-41]. On the other hand, astrocyte activation is not accompanied by proliferation [42-44]. Our present results from CPCP rats showing that spinal microglia were both activated and proliferated, whereas astrocytes were only activated, are consistent with these previous observations in neuropathic pain models. However, Tsuda et al. [20] demonstrated that in the careful observation with proliferation markers, such as Ki-67 and phosphorylated-histone H3 (p-HisH3), spinal astrocyte activation was accompanied by definite proliferation in the neuropathic pain model. Considering this result, there is room for further investigation using such proliferation markers in the CPCP rats.

When L-α-AA, an inhibitor of astrocyte activation, was administered intrathecally at week 5, hyperalgesia in all of the body parts was significantly attenuated, and the activation of the lumbar spinal cord astrocytes that had been activated at this time was also attenuated. These results suggest that activated lumbar spinal cord astrocytes are involved in the maintenance of widespread mechanical hyperalgesia in CPCP rats. Using a complete Freund’s adjuvant-induced inflammation model, Gao et al. [30] showed that heat hyperalgesia occurs only in the inflamed side, whereas mechanical hyperalgesia occurred bilaterally. They also demonstrated that spinal astrocyte activation was involved in mechanical hyperalgesia bilaterally. These results support our present results that astrocyte activation has an important role in the maintenance of mechanical hyperalgesia in the CPCP model. Future research should examine how astrocytes contribute to the molecular mechanisms of pain enhancement.

Lumbar spinal cord microglia were noted to be activated at the onset of mechanical hyperalgesia in the hindpaws in the CPCP model. Coccygeal cord microglia were also activated when mechanical hyperalgesia was present in the tail. With intrathecally administered L-α-AA, all sites of mechanical hyperalgesia in all of the body parts was attenuated; the extent of attenuation in tail hyperalgesia seems to remain at a lower value than the other sites. Additionally, microglial activation in the coccygeal spinal cord was maintained with intrathecal administration of L-α-AA. These results suggest that activated microglia are involved in the onset of hyperalgesia, as well as in the spread of hyperalgesia.

Several studies using nerve injury models have shown activated astrocytes participate in the maintenance of hyperalgesia [2,20,22]. It has been reported that high-density glial activation was observed only in the spinal segments ipsilateral to the injured nerve, and hyperalgesia arose only in the nerve-injured side [20,22,23,25,35-37]. The present findings demonstrate that spinal astrocyte activation in the CPCP model may not accompany clear nerve injury. Our data also indicate that immobilization-induced bilateral glial activation spread beyond the spinal cord segments innervating the immobilized limb, which suggests a different pain inducing mechanism.

The present study showed that hyperalgesia in CPCP rats involved the lumbar and coccygeal spinal segments. This widespread pain is uncharacteristic of neuropathic pain [20,22,25,35-37]. However, we should address if widespread hyperalgesia in our CPCP model is due to whole-leg casting. In other words, in the neuropathic pain model, only specific branches of the sciatic nerves are injured and only the innervating spinal segments are involved. In the CPCP model, however, the entire leg is inserted in a cast and the affected area is larger. It is possible that a larger number of innervating nerves were affected and an increased number of spinal segments were involved, and in this case, the observed phenomenon could be neuropathic. The present experiment, however, showed that ATF3-positive cell counts were small in L3, L4, and L5 DRG of CPCP rats. These results suggest that 2-week cast immobilization of one hindpaw did not cause clear nerve damage.

A recent study from our laboratory suggested the contribution of reactive oxygen species (ROS) produced by ischemia/reperfusion injury to the development of widespread hyperalgesia in CPCP rats [18]. It has been reported that ROS receptors expressed on microglia in the CNS contribute to neuropathic pain [45,46]. Considering these observations, it is reasonable to assume that ROS produced after cast removal directly activate microglia in the CNS. However, the results of that study also showed that an ipsilateral sciatic nerve block performed at the initial appearance of local inflammation (24 h after cast removal) significantly reduced CWP in the CPCP rats. Based on this observation, the results of the present study suggest that ROS sensitizes the primary sensory neurons in the immobilized hindlimb, and consequently activates spinal microglia. This hypothesis is supported by the present result showing that the pERK immunoreactivity in the ipsilateral small DRG cells was increased 1 day after cast removal, implying that the primary nociceptive neurons were activated.

## Conclusions

In the present study, we observed three primary findings. First, immobilization-induced widespread pain is reproducible in rat models. Second, temporal and spatial spreading of activated glial cells is likely involved in the pain mechanism of CPCP. Finally, activation of spinal astrocytes contributes to the maintenance mechanism of widespread hyperalgesia. Elucidating the maintenance mechanism for CWP using the CPCP model has the potential to lead to new therapy strategies for chronic pain.

## Methods

### Animals

The experiments in the present study were conducted with the approval of the Animal Care Committee of Aichi Medical University, and were in accordance with the International Association for the Study of Pain guidelines for pain research in animals. Male Sprague–Dawley rats (300–400 g, Japan SLC, Hamamatsu, Japan) were housed 2–3 per cage under controlled temperature and humidity (23 ± 1°C, 50 ± 15%), a 12-h light/dark cycle, and with free access to food and water. During the 2-week cast immobilization period, the animals were allowed to move about their cages using both forelimbs and the non-immobilized hindlimb. During this period, the levels of general activity and intake of food and water were similar to those before casting. For some experiments, the rats were restrained with a cloth sock from the head to the pelvis. Before the experiments were performed, the animals were habituated to handling and restraint in the sock (1 h daily for 3 days). Once adapted to the process, they typically remained quietly snuggled within the sock, although they were free to emerge; on such occasions, they were gently reintroduced to the restraint. We attest that all efforts were made to minimize the number of animals used and their suffering.

### Hindlimb cast immobilization

CPCP was induced through a 2-week hindlimb cast immobilization, in accordance with the findings of our previous study [18]. In brief, a plaster cast was applied from the trunk to the middle of the left hindpaw under anesthesia with pentobarbital sodium (50 mg/kg, intraperitoneal injection). If signs of circulation impairment (e.g., congestion, ischemia, or pressure ulcer) in the immobilized hindlimb or severe damage to the cast were observed during the 2-week immobility period, the rat was excluded from the behavioral experiments. The remaining animals that completed the 2-week immobility period were restrained with a comfortable cloth sock and their casts were removed by hand. If necessary, scissors also were used. Normal rats were used as controls. The following behavioral tests (including tests before cast removal) were performed as blinded comparative studies.

### Pain behavior

#### Mechanical hyperalgesia

To evaluate mechanical pain behaviors in the hindpaw, each rat was individually placed beneath an inverted plastic box (207 × 132 × 136 mm) with an elevated wire mesh bottom, and was allowed to adjust to the environment for 20 min. From below the mesh floor, a series of calibrated homemade von Frey filaments (VFFs; diameter, 0.5 mm) were applied perpendicularly to the mid-plantar surface of hindpaw. Data were analyzed using the up-and-down method of Dixon and Mood [28]. To investigate mechanical hyperalgesia in the calf skin and tail, as well as pressure stimulation of the calf muscle, the rats were restrained with a sock from the head to the pelvis.

Pressure stimulation of the calf muscle was performed using a Push-Pull Gauge algometer (Aikoh Engineering, Osaka, Japan). A cone-shaped pusher with a rounded tip (diameter, 2.4 mm) was applied to the calf muscle belly with linearly increasing pressure (10 g/s), and the minimum pressure required to elicit foot withdrawal was measured. Pressure stimulation of the calf muscle was performed four times at intervals of at least 30 s, and the median value of the last three trials was defined as the pain threshold. These measurements were also performed on three separate days before the cast was applied, and the average values from these three days were used as baseline control values.

#### Thermal pain behavior

Thermal pain behaviors in both hindpaws were measured with a radiant heat stimulator (Plantar test apparatus, Ugo Basile, Italy). The latency from the onset of radiant heat application to hindpaw withdrawal was defined as the paw withdrawal latency [29]. Before assessing thermal hyperalgesia, the intensity of the radiant heat source was adjusted to yield a mean baseline latency of approximately 10 s for the 11 rats, with an automatic cutoff set at 20 s to avoid tissue damage. The measurements were performed three times with a 5 min interval between each test; the mean value of these three measures was defined as the thermal pain threshold.

### Drug administration

Under isoflurane (2%) anesthesia, a 32-gauge intrathecal catheter (ReCath Co., PA, USA) was inserted through the atlanto-occipital membrane into the lumbar enlargement and externalized through the skin [47]. Four days after catheterization, the catheter placement was verified by the observation of hindlimb paralysis with intrathecal lidocaine injection (2%, 20 μL). Animals that failed to display paralysis with the lidocaine injections were not included in the experiments.

The astroglial toxin L-α-AA was purchased from Sigma (St. Louis, MO, USA). The L-α-AA was dissolved in 0.01 M phosphate-buffered saline (PBS). For L-α-AA administration, doses of 75 nmol (n = 5) and 150 nmol (n = 6) in 20 μL PBS were intrathecally injected at 5 weeks after cast removal, using PBS (20 μL, n = 5) as a vehicle control [22]. After injection, pain behavior was measured at 3 h and 24 h.

### Tissue preparation

The animals were terminally anesthetized with isoflurane (2%) and perfused transcardially through the ascending aorta with 0.1 M PBS (pH 7.4), immediately followed by 4% paraformaldehyde in 0.1 M phosphate buffer (PB, pH 7.4). After the perfusion, the spinal cord segments L4, Co1, and the L3–L5 DRGs were removed and postfixed in the same fixative for 3 h at 4°C, then replaced with 30% sucrose in 0.1 M PB overnight at 4°C. Spinal sections (transverse, free-floating, 30 μm) were cut in a cryostat (Leica CM1850, Nussloch, Germany) and collected in PBS at 4°C or an antifreeze solution (0.05 M sodium phosphate buffer, pH 7.3, containing 30% ethylene glycol and 30% sucrose) and stored at -20°C until they were stained. The DRGs were cut into 14-μm thick longitudinal sections using a cryostat, and were thaw-mounted onto aminosilane-coated glass slides (MAS slide glass; Matsunami Glass Ind., Osaka, Japan), air dried, for processing and stored at -80°C until they were stained.

### Immunohistochemistry

Immunohistochemical staining was conducted with enzymatic or immunofluorescence staining on free-floating sections. The sections were treated with 1% H_2_O_2_ and 40% methanol in Tris-buffered saline (TBS) for 20 min at room temperature to suppress endogenous peroxidase activity. The sections were then incubated in a blocking solution (3% normal goat serum) for 1 h at room temperature. The sections were incubated for 48 h at 4°C with the primary antibodies against cell markers; microglia, OX42 (mouse monoclonal anti-OX42, 1:3000 for enzymatic staining, 1:1000 for immunofluorescence staining; Millipore, Billerica, MA, USA), and astrocytes; GFAP (mouse monoclonal anti-GFAP, 1:5000 for enzymatic staining, 1:2000 for immunofluorescence staining; Millipore, Billerica, MA, USA) and ATF3 (rabbit anti-ATF3, 1:500; Santa Cruz Biotechnologies, CA, USA) were used as markers of cellular stress and injury. For double immunofluorescence, DRG sections were incubated with a mixture of anti-pERK (rabbit monoclonal, neuronal marker, 1:100, Cell Signaling Technology, Beverly, MA, USA) and anti-SP (mouse monoclonal, neuronal marker, 1:1000; R & D Systems, Minneapolis, MN, USA). After primary antibody application, the sections were incubated in secondary antibody (biotinylated goat anti-mouse immunoglobulin G (IgG), 1:500; Jackson ImmunoResearch, West Grove, PA, USA) at room temperature for 2 h. The sections were reacted with an ABC kit (Vector Laboratories, Burlingame, CA, USA) for 1 h, and finally incubated with 0.05% diaminobenzidine and 0.00006% H_2_O_2_. For immunofluorescence labeling, the sections were washed and incubated for 2 h at room temperature with the fluorescent conjugated secondary antibodies (goat anti-mouse IgG-conjugated Alexa Fluor 488 and Alexa Fluor 594, 1:1000; Molecular Probes Life Technologies, Carlsbad, CA, USA). The sections were mounted with Vectashield (Vector Laboratories, Burlingame, CA, USA). Digital images were obtained with a Keyence BIOREVO BZ-9000 microscope (Keyence, Osaka, Japan) and analyzed with image analysis software (Keyence).

### Quantitative image analysis

To quantify positive cell profiles in the spinal cord, five to eight nonadjacent sections from L4 and Co1 spinal cord segments of each rat were randomly selected. Images were captured under a 40× (L4), 60× (Co1), or 10× (L3–5 DRG) objective. Five to six rats were included in each group for quantification. Resting and activated astrocytes or microglia in enzymatic staining (Figure 2) were classified based on the following criteria: resting glia displayed small compact somata bearing long, thin, ramified processes, whereas activated glia exhibited marked cellular hypertrophy (Figure 2C). According to the criteria, cells were sampled only if the nucleus was visible within the plane of section and if cell profiles exhibited distinctly delineated borders [48]. Sampled cells were marked manually by an investigator who was blinded to the treatment [44]. Glial staining by immunofluorescence (Figures 4, 5, and 7) was calculated for the total area of immunoreactivity. The number of ATF3 positive cells (Figure 6, Table 1) was expressed as a percentage of the total number of cells with visible nuclei present in the section. The above-mentioned analyses were performed with image analysis software (Keyence).

### Statistical analysis

Values are presented as the mean ± the standard error of the mean (SEM). The statistical analyses were performed using unpaired *t*-tests, a one-way ANOVA, or a mixed-design two-way ANOVA with repeated measures followed by Dunnett’s test. Differences were considered statistically significant at p < 0.05.

## Abbreviations

ATF3: Activating transcription factor 3; CNS: Central nervous system; CPCP: Chronic post-cast pain; Co1: 1st Coccygeal; CWP: Chronic widespread pain; CRPS: Complex regional pain syndrome; DRG: Dorsal root ganglia; GFAP: Glial fibrillary acidic protein; L-α-AA: L-α-aminoadipate; L4: 4th Lumbar; pERK: Phosphorylated extracellular signal-regulated kinase; p-HisH3: Phosphorylated-histone H3; ROS: Reactive oxygen species; SP: Substance P.

## Competing interests

The authors declare that they have no competing interests.

## Authors’ contributions

MO and YO designed the experiments, analyzed the data, and drafted the manuscript. MO, YO, HS, TY, and AM performed the behavioral tests. MO, YO, HO, and QL performed the immunohistochemical experiments. MO, YO, and JS wrote the manuscript. TN commented on the manuscript and helped to edit the manuscript. JS supervised the experiments, edited the manuscript, and helped interpret the results. All authors have read and approved the final manuscript.
